# Reduced Neointima Formation After Arterial Injury in CD4−/− Mice Is Mediated by CD8^+^CD28^hi^ T Cells

**DOI:** 10.1161/JAHA.113.000155

**Published:** 2013-06-21

**Authors:** Paul C. Dimayuga, Kuang‐Yuh Chyu, Wai Man Lio, Xiaoning Zhao, Juliana Yano, Jianchang Zhou, Tomoyuki Honjo, Prediman K. Shah, Bojan Cercek

**Affiliations:** 1 Oppenheimer Atherosclerosis Research Center, Division of Cardiology, Cedars‐Sinai Heart Institute, Cedars‐Sinai Medical Center, Los Angeles, CA (P.C.D., K.Y.C., W.M.L., X.Z., J.Y., J.Z., T.H., P.K.S., B.C.)

**Keywords:** CD8^+^ T cells, cytolytic activity, neointima, vascular smooth muscle cells

## Abstract

**Background:**

CD8^+^ T‐cell activation, characterized by increased CD28 expression, reduces neointima formation after arterial injury in mice. The CD8^+^CD28^hi^ phenotype is associated with increased effector function. In this study, we used a mouse model that has CD8^+^ but no CD4^+^ T cells (CD4−/−) to assess the role of CD8^+^ T cells and test the function of CD8^+^CD28^hi^ T cells in modulating neointima formation after arterial injury.

**Methods and Results:**

Neointima formation after pericarotid arterial cuff injury was significantly less in CD4−/− mice compared with wild‐type (WT) mice. Negligible baseline lytic activity by splenic CD8^+^ T cells from uninjured WT mice against target syngeneic smooth muscle cells was significantly increased 7 days after injury. Interestingly, CD8^+^ T cells from uninjured CD4−/− mice had significant lytic activity at baseline that remained elevated 7 days after injury. CD8^+^ T‐cell lytic activity was significantly reduced by depletion of CD28^hi^ cells. CD8^+^CD28^hi^ T cells adoptively transferred into recipient Rag‐1−/− mice significantly reduced neointima formation compared with CD8^+^CD28^+^ T‐cell recipient mice.

**Conclusions:**

CD8^+^ T cells reduced neointima formation after arterial injury, attributed in part to increased function of the CD8^+^CD28^hi^ phenotype.

## Introduction

T‐cell activation plays an important role in neointima formation in response to arterial injury.^1–7^ We recently reported that both CD4^+^ and CD8^+^ T cells were activated after arterial injury, as evidenced by increased CD44 expression on both types of T cells. However, subsequent adoptive transfer experiments indicated that only CD8^+^ T cells modulated neointima formation. This was associated with increased CD28 expression in CD8^+^ T cells, but not in CD4^+^ T cells.^8^

Under basal conditions, CD28 is expressed by mouse CD8^+^ T cells (CD8^+^CD28^+^), but is upregulated in a subpopulation on activation (CD8^+^CD28^hi^).^9^ Effector functions after activation have been associated with the CD28^hi^ phenotype.^10–11^ In addition, mouse memory CD8^+^ T cells are subdivided based on CD28 expression into CD28^+^ and CD28^hi^.^12^ Thus, we selected CD28^hi^ as the phenotype marker for further study of CD8^+^ T cells in the response to arterial injury.

CD4 and CD8 T cells contain heterogeneous subpopulations. Specific T‐cell response to arterial injury remains unclear given the lack of understanding of the role of various T‐cell subtypes, highlighted by very few studies on CD8^+^ T cells. In the current report, we investigated the role of CD8^+^ T cells in neointima formation using a mouse model that has CD8^+^ but no CD4^+^ T cells (CD4−/− mice) and compared it with that of wild‐type (WT) mice. This CD4−/− mouse model enabled us to assess the role of CD8^+^ T cells in the absence of helper T cells in the response to arterial injury.^8^ The role of CD8^+^ T cells was further evaluated by testing the function of the CD28^hi^ subpopulation in neointima formation.

Our results confirm the previous report on the role of CD8^+^ T cells in reducing neointima formation^8^ and extend it by identifying the CD8^+^CD28^hi^ as the specific subpopulation involved in this process.

## Methods

### Arterial Injury

Mice were housed in a pathogen‐free facility with access to food and water ad libitum. Aseptic periadventitial cuff injury was performed on the right carotid artery of 25‐week‐old male WT, CD4−/−, or Rag‐1−/− mice on a C57Bl6/J background (Jackson Laboratory), as previously described.^7–8,7–14^ Briefly, mice were anesthetized with ketamine and xylazine, and carprofen was administered prior to surgery for postsurgical pain relief. The right carotid artery was dissected and exposed, a 2.5‐mm‐long Tygon tube (internal diameter of 0.51 mm) with a longitudinal opening was placed around the right carotid artery and secured with ligatures around it, and the wound was closed with sutures. Carotid arteries were harvested after perfusion with normal saline for 10 minutes. The Cedars‐Sinai Institutional Animal Care and Use Committee approved the experimental protocols specifically used in this study.

### Morphometric Analysis

Frozen sections 6 to 8 μm thick were collected from the injured carotid arteries and stained with hematoxylin and eosin or Verhoeff Elastic Van Geison (Sigma), and the vessel area was measured as described previously using image analysis software (ImagePro).^15^

### Flow Cytometry

At different times after injury, mice were euthanized, and the regional lymph nodes and spleens were collected and subjected to red blood cell lysis. Cells were then stained with CD8b and CD44, CD25, or CD28 (BD Bioscience). Mouse aortic smooth muscle cells (SMCs) grown in DMEM/F12 medium supplemented with 10% FBS were trypsinized and stained with monoclonal antibodies to B7‐1 and B7‐2 (eBioscience). Matching IgG isotypes were used as control. Cells were analyzed on a BD LSR‐II apparatus (BD Bioscience).

### T‐Cell Enrichment and Transfer

Spleen cells from age‐matched donor CD4−/− mice were pooled. The cells were then negatively selected for CD8^+^ T cells using a commercially available kit (Invitrogen) with paramagnetic beads and a magnetic particle concentrator,^7–8^ as recommended by the manufacturer (Dynal). The isolated CD8^+^ T cells were further subjected to separation into CD28^+^ and CD28^hi^ using a biotinylated CD28 antibody and paramagnetic beads with a Cellection Biotin Binder kit (Dynal). After magnetic separation of cell‐bound beads, the supernatant was collected as CD28^+^ cells. The cells were then detached from the beads and collected as CD28^hi^ cells. Flow cytometry of CD28 stained cells confirmed the enrichment of the cell populations. An equal number of cells were then injected via the tail vein of recipient Rag‐1−/− mice (2×10^6^ cells/mouse) 48 hours before injury.^7–8,16^ At least 2 separate isolation and transfer procedures were performed.

### Immunohistochemistry

Frozen sections 6 to 8 μm thick were fixed in ice‐cold acetone and stained for active caspase‐3 or CD8b (BD Biosciences). Biotin‐conjugated secondary antibody was used for detection and visualized using horseradish peroxidase–conjugated streptavidin and AEC (DAKO). Omission of the primary antibody was used as a negative control.

### SMC Lytic Assay

The CD8^+^ cytolytic assay was performed as previously described.^8^ Briefly, spleen CD8^+^ T cells negatively purified from mice after euthanasia were cocultured with CFSE‐labeled (4.0 μmol/L) syngeneic aortic SMCs in 10% FBS/DMEM for 4 hours in 37°C at a CD8^+^ T cell to SMC ratio of 3:1. Basal lysis of SMCs without T cells was used as a control. Cells were washed in 1× PBS, trypsinized to detach the SMC monolayer, and stained with 7‐AAD for cell lysis as previously described.^17^ Flow cytometry was performed to detect 7‐AAD‐stained viable SMCs gated on FL1 (CFSE^+^). Gating on CFSE assured viability of the cells that were analyzed. Results are presented as percent SMC lysis.^8^ Depletion of CD28^hi^ cells was performed using biotinylated anti‐mouse CD28 antibody and a CELLection Biotin Binder kit with paramagnetic beads. Flow cytometry of CD28 stained cells confirmed depletion. Perforin blocking was performed using a polyclonal rabbit anti‐perforin antibody (Torrey Pines Biolabs) at a dose of 20 μg/mL.

### Reverse‐Transcription PCR

Individual spleens were collected and TRIzol (Invitrogen) was used to extract RNA. Real‐time polymerase chain reaction (PCR) to assess IL‐2 expression with ubiquitin as a reference was performed using the delta‐delta Ct method. For isolated CD8^+^ T cells, aliquots were taken prior to performing lytic assays and pooled**,** or the culture medium after lytic assays was pooled and collected according to groups and centrifuged to pellet CD8^+^ T cells. RNA was extracted**,** and real‐time PCR was used to assess cytotoxic T‐lymphocyte antigen‐4 (CTLA‐4) expression with ubiquitin as a reference using the delta‐delta Ct method. Carotid arteries were pooled from 4 to 5 mice, the RNA extracted, subjected to reverse transcription using Superscript II (Invitrogen), and PCR performed using B7‐1^18^ and β‐actin^19^ primers. Samples were visualized using agarose gel with ethidium bromide. Densitometric analysis was performed using ImageJ.

### Statistics

Results are presented as mean±standard deviation. Statistical analysis was performed using Prism 3.0. The Student *t* test was used to analyze 2 groups. ANOVA was used to test for significance for multiple groups followed by the Newman–Keuls post test for comparison. Significance was set at *P*<0.05.

## Results

### Characterization of CD8^+^ T Cells in CD4−/− Mice After Arterial Injury

**Figure 1.**
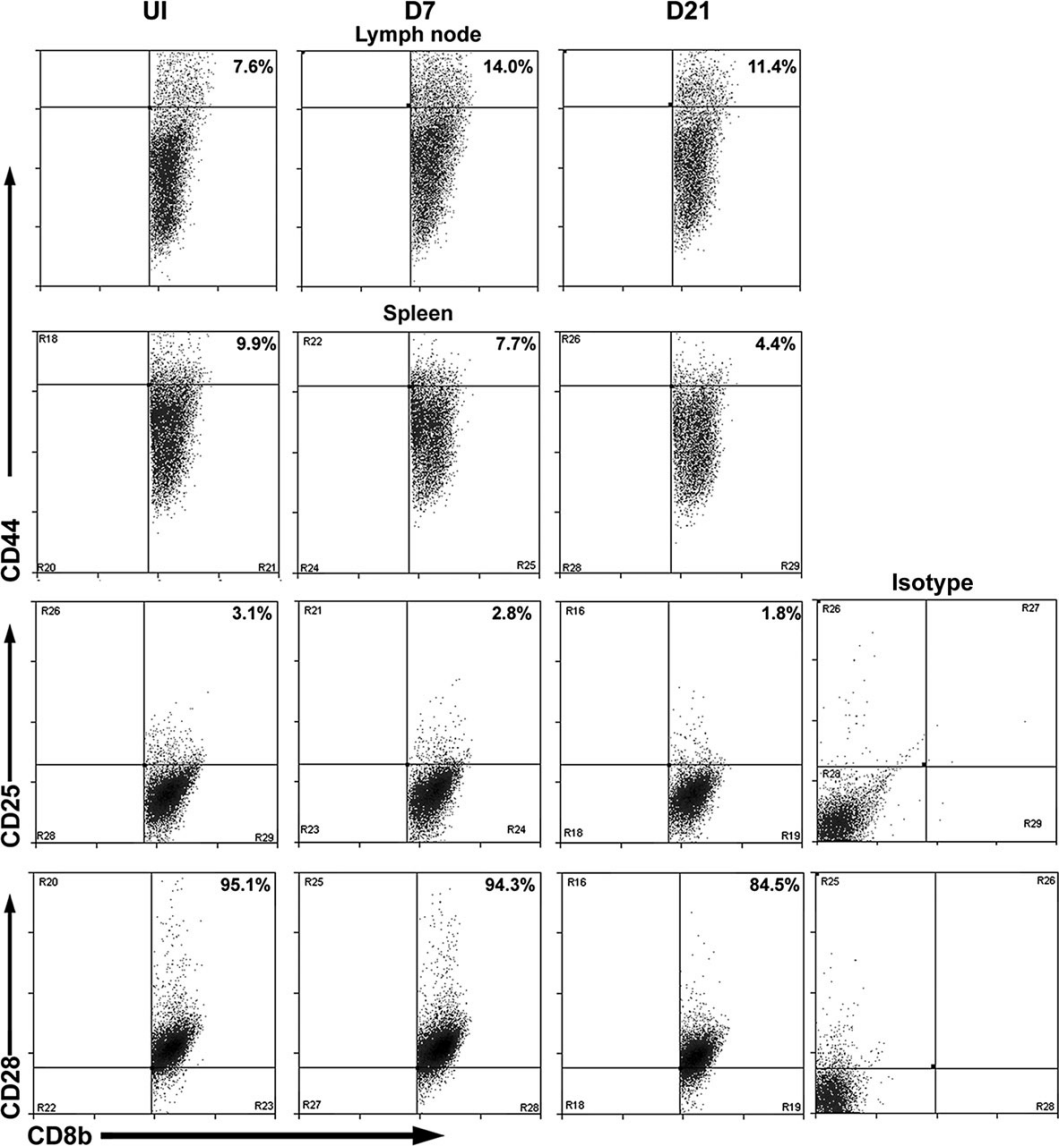
Characterization of CD8^+^ T‐cell response to arterial injury in CD4−/− mice. Representative scatter plots of activation markers CD44, CD25, and CD28 assessed in lymph node (top row) and spleen (second, third, and bottom rows) in uninjured mice (UI, left column) or 7 and 21 days after injury (D7 and D21, middle and right columns, respectively). The CD8b^+^ (FITC) gate was used for cell analysis. CD44, CD25, and CD28 antibodies were PE conjugated. FITC indicates fluorescein isothiocyanate; PE, phycoerythrin.

Arterial injury in WT mice results in T‐cell activation characterized by increased CD28 expression on CD8^+^ T cells that have the propensity to reduce neointima formation.^8^ Thus, we characterized CD8^+^ T‐cell response to injury in mice that have CD8^+^ T cells but not CD4^+^ T cells. Spleen and lymph nodes were collected from uninjured or 7‐ and 14‐day injured CD4−/− mice. There was a slight but significant increase in CD8^+^CD44^+^ T cells in lymph nodes of CD4−/− mice 7 days after injury (Figure 1, top).

However, in contrast to the temporal changes of phenotypes after injury in WT mice,^8^ splenic CD8^+^ T cells of CD4−/− mice had significantly reduced CD8^+^CD44^hi^ T cells (Figure 1, second panel), CD8^+^CD25^+^ T cells (Figure 1, third panel), and CD8^+^CD28^+^ T cells (Figure 1, bottom) 21 days after arterial injury (Table 1). Thus, the profile observed in the injured CD4−/− mice did not correspond with that observed in injured WT mice.^8^ Neointima formation after injury was then compared between WT and CD4−/− mice.

**Table 1.** CD8^+^ T‐Cell Profile After Arterial Injury in CD4−/− Mice

	No Injury	Day 7	Day 21
LN CD8^+^CD44^hi^	9.7±3.2(n=4)	16.1±2.6* (n=3)	11.7±2.5 (n=4)
Spln CD8^+^CD44^hi^	8.5±0.9 (n=4)	8.6±1.5 (n=3)	4.7±1.7* (n=6)
Spln CD8^+^CD25^+^	3.2±0.3 (n=5)	3.1±0.1(n=8)	2.1±0.4* (n=8)
Spln CD8^+^CD28^+^	94.0±3.4 (n=7)	90.6±2.2* (n=7)	87.4±2.0** (n=8)

All values are percentage of CD8^+^‐gated cells±standard deviation. LN indicates lymph node; Spln, spleen.

* *P*<0.05 vs no injury.

* *P*<0.01 vs no injury.

* *P*<0.05 vs day 7.

* *P*<0.001 vs no injury.

### Reduced Neointima Formation in CD4−/− Mice Compared With WT Mice

**Figure 2.**
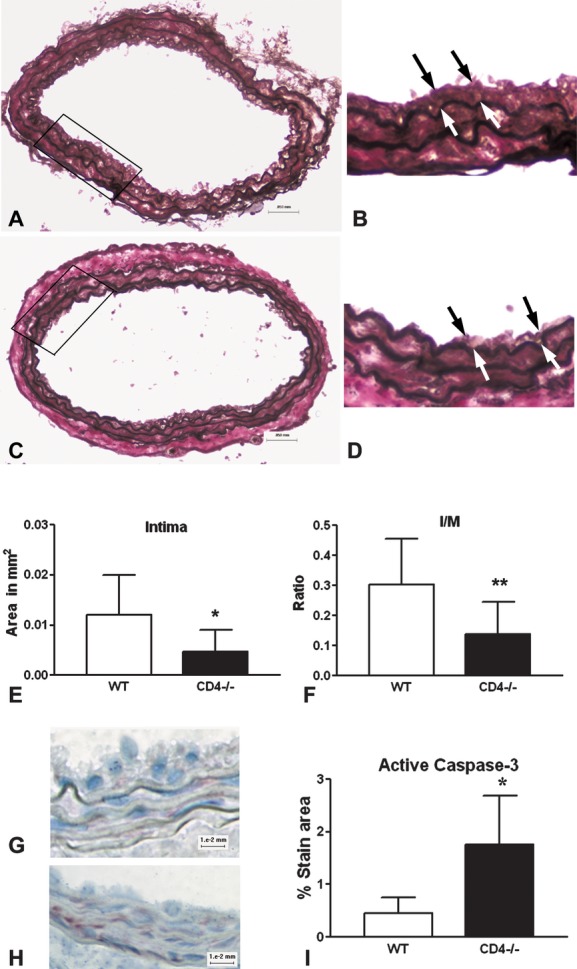
Neointima formation after arterial injury. Representative sections with elastin Von Geison stain 21 days after injury in wild‐type (WT) (A and B) and CD4−/− (C and D) mice. Bar graph of area measurements for intima (E) and intima‐to‐media (I/M) ratio (F). *P=0.01; **P=0.007; WT, n=12; CD4−/−, n=11. Bar=50 μm; (B) and (D) are ×40 magnification. Representative sections stained for active caspase‐3 from WT (G) and CD4−/− (H) mice. Bar=10 μm. Histomorphometric analysis of percent active caspase‐3‐stained area (I). *P=0.04; n=4 each.

Twenty‐one days after arterial injury there was significantly reduced neointima formation in CD4−/− mice compared with WT mice (0.005±0.004 mm^2^, n=11 versus 0.012±0.008 mm^2^, n=12, respectively; *P*=0.01; Figure 2A through 2E); whereas no difference was observed in the medial area. Hence, intima‐to‐media ratio (I/M) was significantly reduced in CD4−/− mice compared with WT mice (0.14±0.03 versus 0.30±0.04, respectively; *P*=0.007; Figure 2F). This reduced neointima formation in CD4−/− mice was associated with significantly increased active caspase‐3 immunoreactivity in the injured arteries of CD4−/− mice compared with WT mice (Figure 2G through 2I), suggesting increased SMC death in CD4−/− mice.

### Cytolytic Activity of CD8^+^ T Cells From WT and CD4−/− Mice

**Figure 3.**
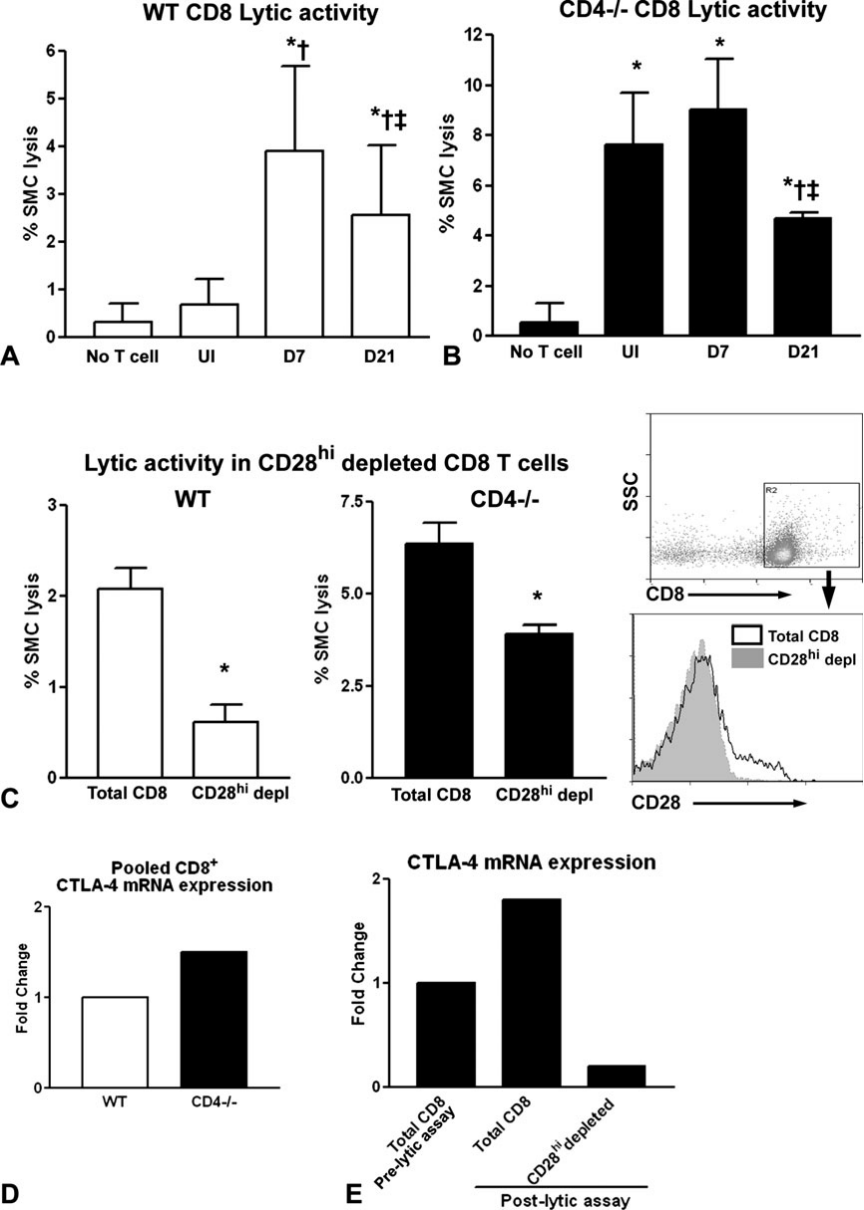
CD8^+^ T‐cell cytolytic activity against syngeneic SMCs. CD8^+^ T cells were negatively isolated from spleens of uninjured (UI) WT (A) or CD4−/− (B) mice or 7 (D7) and 21 (D21) days after injury and cultured with target SMCs in a T‐cell to target ratio of 3:1. A, WT, n=8 to 17; *P<0.001 vs no T cells; †*P*<0.001 vs UI; ‡*P*<0.01 vs D7. B, CD4−/−, n=4 to 7; *P<0.001 vs no T cells; †*P*<0.01 vs UI; ‡*P*<0.01 vs D7. Cytolytic activity of CD8^+^ T cells after depletion of CD28^hi^ from WT mice 7 days after injury (C, left) and uninjured CD4−/− mice (C, middle). Right panel shows histogram of CD8b^+^‐gated cells after CD28^hi^ depletion. CD8b antibody is PE conjugated, and CD28 antibody is APC conjugated. WT, n=3 each; *P=0.001; CD4−/−, n=4 each; *P=0.002. CTLA‐4 mRNA expression was assessed in isolated CD8^+^ T cells pooled from WT or CD4−/− mice before lytic assays (D) and in CD8^+^ T cells pooled after lytic assays in whole CD8^+^ or CD28^hi^‐depleted CD8^+^ cells from CD4−/− mice (E). SMC indicates smooth muscle cell; WT, wild type; CTLA‐4, cytotoxic T‐lymphocyte antigen‐4; APC, allophycocyanin.

Given the increased active caspase‐3 immunoreactivity in the injured arteries of CD4−/− mice, we hypothesized that vascular smooth muscle cells are targets of activated CD8^+^ T cells after injury. The cytolytic activity of CD8^+^ T cells from spleens of CD4−/− and WT mice against syngeneic aortic SMCs as target cells was therefore assessed at the different points after injury. SMC lysis by CD8^+^ T cells from uninjured WT mice was negligible (Figure 3A). There was a significant increase in SMC lysis by CD8^+^ T cells from 7‐day injured mice, which persisted to a lesser degree in 21‐day injured mice. In contrast, CD8^+^ T cells from uninjured CD4−/− mice (Figure 3B) already had significant lytic activity at baseline, which further increased slightly 7 days after injury and significantly decreased at day 21.

### CD4−/− Mice Have Increased IL‐2 Expression in the Steady State

Cytolytic activity of CD8^+^ T cells is enhanced by IL‐2.^20–21^ Hence, we hypothesized that the higher lytic activity of CD8^+^ T cells from uninjured CD4−/− mice compared with WT mice is a result of higher splenic steady‐state IL‐2 expression in CD4−/− mice. Splenic IL‐2 mRNA expression was therefore assessed between uninjured WT and CD4−/− mice in the steady state. There was significantly higher IL‐2 expression in the CD4−/− mice (n=4) compared with WT mice (n=7; 2.1±0.8 versus 0.9±0.5 fold‐change, respectively; *P*=0.01).

### CD8^+^CD28^hi^ T Cells Have Lytic Function

The increased lytic activity in CD8^+^ T cells from 7‐day injured WT mice parallels our previously reported increase in CD28 expression 7 days after injury.^8^ Given CD8^+^ T cells from D7 resulted in the highest lytic activity against VSMC, we chose this time to determine if the CD28^hi^ cells are involved in the SMC lytic activity of CD8^+^ T cells from WT mice. CD28^hi^ cells were depleted from pooled CD8^+^ T cells, and the lytic activity was assessed on the remaining cells. Depletion of CD28^hi^ cells from the CD8^+^ T cell pool of 7‐day injured WT mice significantly reduced cytolytic activity against SMCs (Figure 3C, left).

Because lytic activity was already high in uninjured CD4−/− mice, CD28^hi^ depletion was also performed with CD8^+^ T cells pooled from uninjured CD4−/− mice and the cells tested for lytic activity. Depletion of CD28^hi^ cells from the CD8^+^ T cells pooled from uninjured CD4−/− mice significantly reduced cytolytic activity against SMCs (Figure 3C, middle). Flow‐cytometric analysis confirmed the depletion of CD28^hi^ cells (Figure 3C, right).

### Increased Lytic Activity Coincides With Increased CTLA‐4 Expression

To determine if the opposing signaling molecule was also present in CD8^+^ T cells that had increased cytolytic activity, we assessed CTLA‐4 mRNA expression from CD8^+^ T cells pooled from spleens of uninjured WT or CD4−/− mice aliquoted prior to the lytic assays. There was higher CTLA‐4 expression observed in the CD8^+^ T cells from CD4−/− mice (Figure 3D).

CTLA‐4 expression was then assessed in the total CD8^+^ T‐cell pool from CD4−/− mice before and after the cytolytic assay. Culture medium was collected after several lytic assays and centrifuged to pellet the CD8^+^ T cells. Cell pellets were then pooled to assay for CTLA‐4 mRNA expression. There was a nearly 2‐fold increase in CTLA‐4 expression in total CD8^+^ T cells after the cytolytic assay. This occurred primarily in the CD28^hi^ population because CD8^+^ T cells depleted of CD28^hi^ cells did not have increased CTLA‐4 mRNA expression (Figure 3E).

### Arterial SMCs Express the CD28 Ligand B7‐1

**Figure 4.**
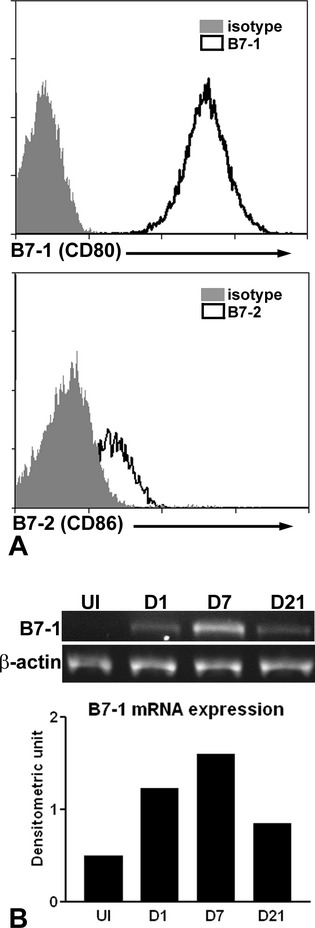
Expression of costimulatory molecules on smooth muscle cells (SMCs). Histogram depicting fluorescent staining of SMCs for B7‐1 (A, top) and B7‐2 (A, bottom). Representative of 3. B7‐1 antibody is allophycocyanin (APC) conjugated and B7‐2 antibody is Alexa Fluor 700 conjugated. Reverse‐transcription polymerase chain reaction (RT‐PCR) of injured carotid arteries from wild‐type (WT) mice for B7‐1 mRNA expression (B, top) with β‐actin as reference gene. Bar graph of densitometric analysis of signal intensity relative to reference gene (B, bottom). Right carotid arteries pooled from 4 to 5 mice per time‐point.

Expression of the CD28 ligand B7‐1 (CD80) increases susceptibility of syngeneic target cells to CD8^+^ T‐cell lysis.^22^ Hence, cultured WT SMCs were checked for expression of known CD28 ligands B7‐1 and B7‐2 (CD86) using flow cytometry and assessed using mean fluorescence intensity (MFI). Significant B7‐1 expression was detected on cultured SMCs (MFI=518.7±6.2, n=3; Figure 4A, top), but B7‐2 was only minimally expressed (MFI=11.5±0.2, n=3; Figure 4A, bottom). To confirm the in vivo relevance of the finding, carotid arteries from 4 to 5 WT mice were pooled and assessed for B7‐1 mRNA expression after injury at different times. Minimal B7‐1 mRNA was present in uninjured arteries, but increased within a day after injury and peaked 7 days after injury. Low expression of B7‐1 was still detectable 21 days after injury (Figure 4B).

### CD8^+^CD28^hi^ T Cells Reduce Neointima Formation

**Figure 5.**
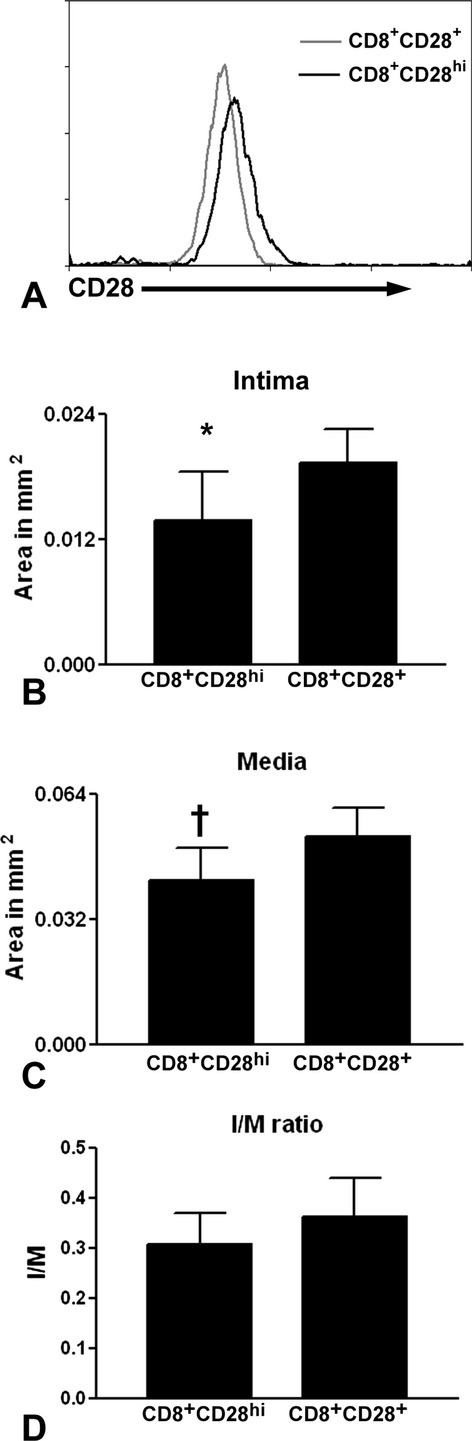
Neointima formation after adoptive cell transfer into recipient Rag‐1−/− mice. Histogram of CD8b‐gated CD28^+^ and CD28^hi^ cells (A). CD8b antibody is fluorescein isothiocyanate (FITC) conjugated and CD28 antibody is PE conjugated. Neointima (B), media (C), and intima‐to‐media (I/M) ratio (D) measurements of carotid arteries of recipient mice 21 days after injury. Neointima *P=0.01; media †*P*=0.007; CD28^hi^, n=8; CD28^+^, n=10.

We then used adoptive transfer strategy to test the functional role of the CD28^hi^ population of CD8^+^ T cells from CD4−/− mice in reducing neointima formation in injured arteries. CD8^+^ T cells enriched for or depleted of CD28^hi^ cells from donor CD4−/− mice were adoptively transferred into immune‐deficient Rag‐1−/− mice (CD8^+^CD28^hi^ or CD8^+^CD28^+^ recipients, respectively). Flow‐cytometric analysis of the MFI verified enrichment of CD28^hi^ T cells from the donor CD8^+^ T‐cell pool (CD8^+^CD28^hi^ MFI=84.8, CD8^+^CD28^+^ MFI=40.5; Figure 5A). It should be noted that the majority of mouse CD8^+^ T cells express CD28, and the distinction between CD28^+^ and CD28^hi^ is based on separation between cells that have background levels of CD28 and cells that have increased expression, respectively, as determined by MFI.^23^ Recipients of CD8^+^CD28^hi^ T cells had significantly reduced neointima formation compared with recipients of CD8^+^CD28^+^ T cells (Figure 5B). Medial area was also significantly less in the CD8^+^CD28^hi^ T‐cell recipient mice compared with the CD8^+^CD28^+^ recipient mice (Figure 5C). Intima:media (I/M) ratio was trending less in the CD8^+^CD28^hi^ recipient mice compared with CD8^+^CD28^+^ T‐cell recipient mice (Figure 5D) but was not statistically different (*P*=0.1).

### Adoptively Transferred CD8^+^ T Cells Localize to the Injured Arterial Wall

**Figure 6.**
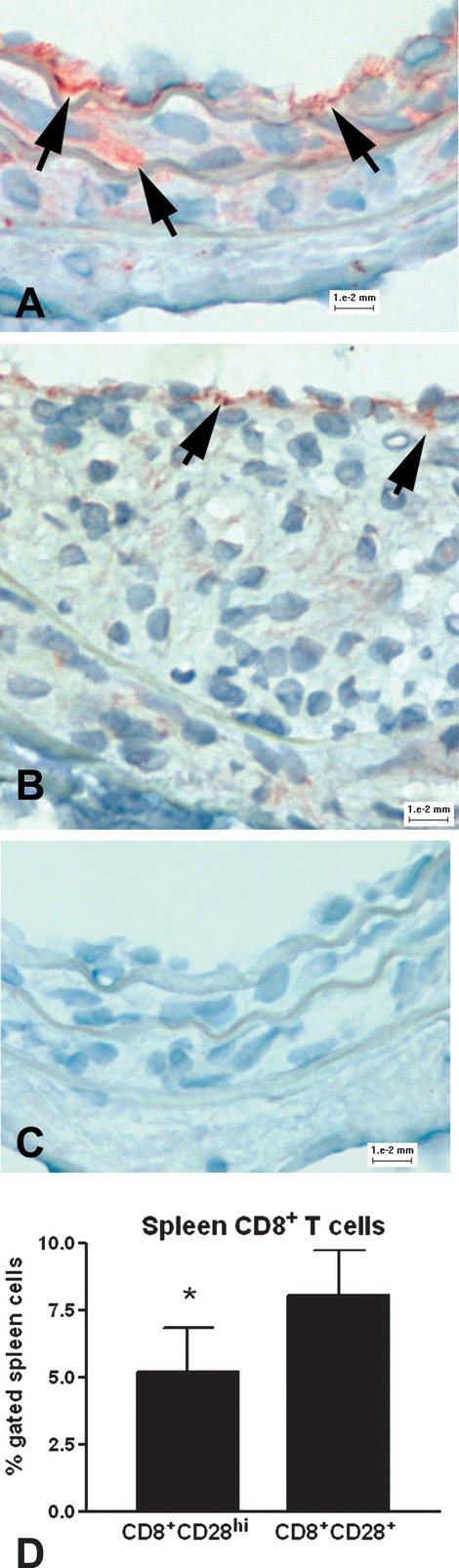
Homing of adoptively transferred CD8^+^ T cells. Representative staining of CD8b^+^ T cells in the injured arteries of the recipient mice that received CD8^+^CD28^hi^ (A) or CD8^+^CD28^+^ (B) cells. Omission of primary antibody was used as a control (C). Arrows indicate positive staining. Bar=0.01 mm. Flow‐cytometric analysis also showed the presence of viable CD8^+^ T cells in the spleens of both recipient groups (D). CD8^+^CD28^hi^, n=6; CD8^+^CD28^+^, n=9; *P=0.007.

CD8^+^ T‐cell homing to the injured arterial wall was determined using immunostaining of cross sections from both the CD8^+^CD28^hi^ recipient mice and the CD8^+^CD28^+^ T‐cell recipient mice. There were more CD8^+^ T cells localized in the arteries of the CD8^+^CD28^hi^ recipient mice compared with the CD8^+^CD28^+^ T‐cell recipient mice (Figure 6A through 6C). Histomorphometric analysis showed a significantly increased percentage of CD8^+^ T‐cell stained area in the CD8^+^CD28^hi^ recipients compared with the CD8^+^CD28^+^ T‐cell recipient mice (2.5±0.3% versus 1.6±0.6%, respectively; n=4 each; *P*=0.04). CD8^+^ T cells were also detected in the spleens of both recipient groups 21 days after injury, but there were significantly more in the CD8^+^CD28^+^ recipient mice (Figure 6D).

## Discussion

In the current study, we have not only confirmed our previous observation that CD8^+^ T cells reduce neointima formation after arterial injury by testing it in a different model (CD4−/− mice), but also provided the following novel findings: (1) the CD28^hi^ subpopulation of CD8^+^ T cells are involved in the response to arterial injury; (2) cytolytic activity against syngeneic SMCs is predominantly by the CD28^hi^ subpopulation of CD8^+^ T cells; (3) the role of CD8^+^CD28^hi^ T cells in neointima formation using adoptive cell transfer is confirmed; and (4) the CD28 ligand B7‐1 is expressed on smooth muscle cells.

Our previous study showed that CD8^+^ T cells are involved in T‐cell‐mediated reduction of neointima formation after arterial injury. Thus, we explored further the role of CD8^+^ T cells by performing injury in mice that have only CD8^+^, not CD4^+^, T cells (CD4−/− mice). The CD8^+^ T‐cell response to injury was first characterized in the CD4−/− mice. Similar to WT mice,^8^ lymph node CD8^+^CD44^hi^ T cells increased after arterial injury in the CD4−/− mice. However, in contrast to WT mice, splenic CD8^+^ T cells did not show increased activation phenotype after arterial injury. Despite this, CD4−/− mice had significantly reduced neointima formation, confirming our previous report on the role of CD8^+^ T cells in controlling neointima formation.^8^

CD8^+^ T cells function as cytolytic lymphocytes, and we attributed the reduced neointima formation by CD8^+^ T cells partially to CD8^+^ T‐cell lytic activity against syngeneic SMCs.^8^ Hence, the kinetics of lytic activity of splenic CD8^+^ T cells was assessed after arterial injury. In WT mice, we observed increased CD8^+^ T‐cell cytolytic activity 7 days after arterial injury, which persisted to a lesser degree up to 21 days after injury.

Significantly reduced neointima formation in the CD4−/− mice predicted that cytolytic activity would be higher in the CD8^+^ T cells from these mice if, as we hypothesized, CD8^+^ T‐cell‐mediated cytolytic activity was involved in controlling the neointima. Indeed, the profile observed in the CD8^+^ T cells from the CD4−/− mouse spleens differed dramatically from WT mice in that even before arterial injury, there was already significant lytic activity against syngeneic SMCs. The presence of significant lytic activity in the steady (uninjured) state of CD4−/− mice prompted us to assess differences in the immune profile compared with WT mice. IL‐2 is a known regulator of T‐cell function. Relevantly to our studies, exogenous IL‐2 enhanced CD8^+^ cytolytic activity.^20–21^ Thus, increased splenic IL‐2 mRNA expression in the steady state of CD4−/− mice compared with WT mice is in agreement with the reported role of IL‐2 bioavailability in enhancing cytolytic activity.

The results from the injury time course of cytolytic activity of CD8^+^ T cells from WT mice paralleled the increased CD28 expression on WT CD8^+^ T cells after injury that we previously reported,^8^ suggesting that CD28^hi^ may be a phenotypic marker of functional significance. This was confirmed by depleting CD28^hi^ from the CD8^+^ T cells of 7‐day injured WT mice, which resulted in reduced cytolytic activity against syngeneic SMCs.

The signaling that occurs through CD28 during T‐cell activation is downregulated by CTLA‐4. Interestingly, in our study, even as there was increased cytolytic activity by CD8^+^ T cells from CD4−/− mice, there was also concomitantly increased CTLA‐4 mRNA expression. In addition, lytic activity occurred with a compensatory increase in CTLA‐4 expression in the CD8^+^CD28^hi^ T cells. The results suggest that even as the CD28^hi^ subpopulation has increased activity, there remains a measure of self‐control against an overly aggressive response against syngeneic cells.

The role of CD28 in the cytolytic activity by CD8^+^ T cells has been previously reported.^10^ Of particular interest is the finding that increased B7‐1 expression induced susceptibility of syngeneic target cells to CD8^+^ T‐cell lysis.^22^ B7 molecules are known ligands of CD28 and are classic costimulatory molecules. What was unknown is whether B7 molecules are expressed by SMCs. Fluorescent staining and flow‐cytometric analysis showed the presence of B7‐1 on the surface of cultured mouse SMCs. In addition, although minimally expressed in native arteries, B7‐1 mRNA expression was detected after injury of the right carotid artery. Evidence shows that injury to the arterial wall leads to phenotypic changes of vascular smooth muscle cells.^24–25^ Thus, phenotypic changes that occur in both the CD8^+^ T cells and SMCs of the injured artery may contribute to the observed control of neointima formation by CD8^+^CD28^hi^ T cells. That CD28^hi^ is a functional CD8^+^ T‐cell phenotype was further confirmed by adoptive transfer experiments of CD8^+^ T cells that were enriched for CD28^hi^ cells into immune‐deficient Rag‐1−/− mice. CD28^hi^ recipients had significantly reduced neointima formation compared with the CD28^+^ recipients, likely through increased cytolytic activity against syngeneic SMCs.

The increased target cell susceptibility by increased B7‐1 expression noted in the report cited above^22^ was attributed to promiscuous lysis by CD8^+^ T cells and may be MHC‐I nonresticted. It remains unknown if the lytic activity against syngeneic SMCs is MHC‐I restricted, and our previous report suggests that it may not be.^8^ In addition, attempts to block lytic activity using antibody against perforin in our study were unsuccessful (not shown), suggesting that the lytic activity is not mediated by perforin. The implications of our findings combined with other reports are apparent in the potential for autoimmune complications in the vascular wall. It is not unreasonable to speculate that autoimmune‐mediated vasculitis may involve, among other causes, the convergence of changes in the vascular wall and in specific T‐cell subtypes that may not necessarily have antigen specificity. In addition, it remains unknown what role, if any, cytolytic CD8^+^CD28^hi^ T cells may play in arteries that have established atherosclerotic plaques with SMC‐rich fibrous caps. CD8^+^ T‐cell activation precedes CD4^+^ T‐cell activation induced by feeding apoE−/− mice with an atherogenic diet.^26^ Within the CD8^+^ T‐cell population reported in the study, the CD28^+^ subtype had increased activation,^26^ emphasizing the relevance of our results. In addition, our group recently reported the role of CD8^+^ T cells in an apoB‐100‐derived peptide vaccine that reduced atherosclerosis in apoE−/− mice.^27^ Combined with these reports, our study underscores the work ahead to further reveal and better understand the interaction between CD8^+^ T cells and vascular cells.

The limitations of the study include the use of the CD4−/− mice, which have been reported to have compensatory changes.^28^ However, functions attributed to CD8^+^ T cells from the CD4−/− mice were also present in the WT CD8^+^ T cells after arterial injury. The specific difference was that the phenotype and functions of CD8^+^ T cells were exaggerated in the CD4−/− mice, with significantly reduced neointima formation, making it advantageous for our study.

In conclusion, our results provide additional evidence that CD8^+^ T cells are involved in controlling neointima formation after arterial injury. We identified CD8^+^CD28^hi^ T cells as the specific subtype involved. These cells have cytolytic activity against syngeneic SMCs that express the costimulatory molecule B7‐1. This study adds to the growing body of evidence that supports immune regulation of the remodeling of the vascular wall.

## References

[1] Dimayuga PC Chyu KY Cercek B Immune responses regulating the response to vascular injury. Curr Opin Lipidol. 2010; 21:416-421 20613513 10.1097/MOL.0b013e32833cacbe

[2] Zernecke A Liehn EA Gao JL Kuziel WA Murphy PM Weber C Deficiency in CCR5 but not CCR1 protects against neointima formation in atherosclerosis‐prone mice: involvement of IL‐10. Blood. 2006; 107:4240-4243 16467202 10.1182/blood-2005-09-3922

[3] Kovacic JC Gupta R Lee AC Ma M Fang F Tolbert CN Walts AD Beltran LE San H Chen G St HC Boehm M Stat3‐dependent acute Rantes production in vascular smooth muscle cells modulates inflammation following arterial injury in mice. J Clin Invest. 2010; 120:303-314 20038813 10.1172/JCI40364 PMC2798694

[4] Kurobe H Urata M Ueno M Ueki M Ono S Izawa‐Ishizawa Y Fukuhara Y Lei Y Ripen AM Kanbara T Aihara K Ishizawa K Akaike M Gonzalez FJ Tamaki T Takahama Y Yoshizumi M Kitagawa T Tomita S Role of hypoxia‐inducible factor 1alpha in T cells as a negative regulator in development of vascular remodeling. Arterioscler Thromb Vasc Biol. 2010; 30:210-217 20007912 10.1161/ATVBAHA.109.192666 PMC6392182

[5] Hansson GK Holm J Holm S Fotev Z Hedrich HJ Fingerle J T lymphocytes inhibit the vascular response to injury. Proc Natl Acad Sci USA. 1991; 88:10530-10534 1961717 10.1073/pnas.88.23.10530 PMC52962

[6] Zhu B Reardon CA Getz GS Hui DY Both apolipoprotein E and immune deficiency exacerbate neointimal hyperplasia after vascular injury in mice. Arterioscler Thromb Vasc Biol. 2002; 22:450-455 11884289 10.1161/hq0302.105377

[7] Dimayuga PC Li H Chyu KY Fredrikson GN Nilsson J Fishbein MC Shah PK Cercek B T cell modulation of intimal thickening after vascular injury: the bimodal role of IFN‐gamma in immune deficiency. Arterioscler Thromb Vasc Biol. 2005; 25:2528-2534 16224059 10.1161/01.ATV.0000190606.41121.00

[8] Dimayuga PC Chyu KY Kirzner J Yano J Zhao X Zhou J Shah PK Cercek B Enhanced neointima formation following arterial injury in immune Deficient rag‐1−/− mice is attenuated by adoptive transfer of CD8 T cells. PLoS ONE. 2011; 6:e20214 21629656 10.1371/journal.pone.0020214 PMC3101237

[9] Turka LA Ledbetter JA Lee K June CH Thompson CB CD28 is an inducible T cell surface antigen that transduces a proliferative signal in CD3+ mature thymocytes. J Immunol. 1990; 144:1646-1653 2155264

[10] Makrigiannis AP Musgrave BL Hoskin DW Differential effects of B7–1 and B7–2 on the costimulation of mouse nonspecific cytotoxic T lymphocyte development in response to anti‐CD3 antibody. J Leukoc Biol. 1999; 66:792-802 10577511 10.1002/jlb.66.5.792

[11] Butler JJ Mader JS Watson CL Zhang H Blay J Hoskin DW Adenosine inhibits activation‐induced T cell expression of CD2 and CD28 co‐stimulatory molecules: role of interleukin‐2 and cyclic AMP signaling pathways. J Cell Biochem. 2003; 89:975-991 12874832 10.1002/jcb.10562

[12] Ortiz‐Suarez A Miller RA A subset of CD8 memory T cells from old mice have high levels of CD28 and produce IFN‐gamma. Clin Immunol. 2002; 104:282-292 12217339 10.1006/clim.2002.5221

[13] Chyu KY Dimayuga P Zhu J Nilsson J Kaul S Shah PK Cercek B Decreased neointimal thickening after arterial wall injury in inducible nitric oxide synthase knockout mice. Circ Res. 1999; 85:1192-1198 10590247 10.1161/01.res.85.12.1192

[14] Dimayuga P Zhu J Oguchi S Chyu KY Xu XO Yano J Shah PK Nilsson J Cercek B Reconstituted HDL containing human apolipoprotein A‐1 reduces VCAM‐1 expression and neointima formation following periadventitial cuff‐induced carotid injury in apoE null mice. Biochem Biophys Res Commun. 1999; 264:465-468 10529386 10.1006/bbrc.1999.1278

[15] Dimayuga PC Cesena FH Chyu KY Yano J Amorn A Fishbein MC Shah PK Cercek B Natural antibodies and complement modulate intimal thickening after arterial injury. Am J Physiol Regul Integr Comp Physiol. 2009; 297:R1593-R1600 19776252 10.1152/ajpregu.00114.2009 PMC3774189

[16] Dimayuga P Cercek B Oguchi S Fredrikson GN Yano J Shah PK Jovinge S Nilsson J Inhibitory effect on arterial injury‐induced neointimal formation by adoptive B‐cell transfer in Rag‐1 knockout mice. Arterioscler Thromb Vasc Biol. 2002; 22:644-649 11950704 10.1161/01.atv.0000012455.62765.bf

[17] Lecoeur H Fevrier M Garcia S Riviere Y Gougeon ML A novel flow cytometric assay for quantitation and multiparametric characterization of cell‐mediated cytotoxicity. J Immunol Methods. 2001; 253:177-187 11384679 10.1016/s0022-1759(01)00359-3

[18] Peterson KE Sharp GC Tang H Braley‐Mullen H B7.2 has opposing roles during the activation versus effector stages of experimental autoimmune thyroiditis. J Immunol. 1999; 162:1859-1867 9973452

[19] Lee TS Yen HC Pan CC Chau LY The role of interleukin 12 in the development of atherosclerosis in ApoE‐deficient mice. Arterioscler Thromb Vasc Biol. 1999; 19:734-742 10073981 10.1161/01.atv.19.3.734

[20] Hidalgo LG Urmson J Halloran PF IFN‐gamma decreases CTL generation by limiting IL‐2 production: a feedback loop controlling effector cell production. Am J Transplant. 2005; 5:651-661 15760388 10.1111/j.1600-6143.2005.00761.x

[21] Janas ML Groves P Kienzle N Kelso A IL‐2 regulates perforin and granzyme gene expression in CD8+ T cells independently of its effects on survival and proliferation. J Immunol. 2005; 175:8003-8010 16339537 10.4049/jimmunol.175.12.8003

[22] Nishio M Spielman J Lee RK Nelson DL Podack ER CD80 (B7.1) and CD54 (intracellular adhesion molecule‐1) induce target cell susceptibility to promiscuous cytotoxic T cell lysis. J Immunol. 1996; 157:4347-4353 8906809

[23] Menager‐Marcq I Pomie C Romagnoli P van Meerwijk JP CD8+CD28‐ regulatory T lymphocytes prevent experimental inflammatory bowel disease in mice. Gastroenterology. 2006; 131:1775-1785 17087950 10.1053/j.gastro.2006.09.008 PMC1950262

[24] Kocher O Gabbiani F Gabbiani G Reidy MA Cokay MS Peters H Huttner I Phenotypic features of smooth muscle cells during the evolution of experimental carotid artery intimal thickening. Biochemical and morphologic studies. Lab Invest. 1991; 65:459-470 1921335

[25] Oguchi S Dimayuga P Zhu J Chyu KY Yano J Shah PK Nilsson J Cercek B Monoclonal antibody against vascular cell adhesion molecule‐1 inhibits neointimal formation after periadventitial carotid artery injury in genetically hypercholesterolemic mice. Arterioscler Thromb Vasc Biol. 2000; 20:1729-1736 10894810 10.1161/01.atv.20.7.1729

[26] Kolbus D Ramos OH Berg KE Persson J Wigren M Bjorkbacka H Fredrikson GN Nilsson J CD8+ T cell activation predominate early immune responses to hypercholesterolemia in Apoe(−/−) mice. BMC Immunol. 2010; 11:58 21126329 10.1186/1471-2172-11-58 PMC3003229

[27] Chyu KY Zhao X Dimayuga PC Zhou J Li X Yano J Lio WM Chan LF Kirzner J Trinidad P Cercek B Shah PK CD8 T cells mediate the athero‐protective effect of immunization with an ApoB‐100 peptide. PLoS ONE. 2012; 7:e30780 22347402 10.1371/journal.pone.0030780 PMC3276497

[28] Tyznik AJ Sun JC Bevan MJ The CD8 population in CD4‐deficient mice is heavily contaminated with MHC class II‐restricted T cells. J Exp Med. 2004; 199:559-565 14769854 10.1084/jem.20031961 PMC2211827

